# Increasing Acid Concentration, Time and Using a Two-Part Silane Potentiates Bond Strength of Lithium Disilicate–Reinforced Glass Ceramic to Resin Composite: An Exploratory Laboratory Study

**DOI:** 10.3390/ma15062045

**Published:** 2022-03-10

**Authors:** Matilde Almiro, Beatriz Marinho, António H. S. Delgado, João Rua, Paulo Monteiro, Inês Caetano Santos, Luís Proença, José João Mendes, Marco M. M. Gresnigt

**Affiliations:** 1 Department of Conservative Dentistry, Instituto Universitário Egas Moniz (IUEM), Monte de Caparica, 2829-511 Almada, Portugal; matildealmiro.md@gmail.com (M.A.); beatriz_marinho@hotmail.com (B.M.); 2 Centro de Investigação Interdisciplinar Egas Moniz (CiiEM), Instituto Universitário Egas Moniz (IUEM), Monte de Caparica, 2829-511 Almada, Portugal; aldelgado@egasmoniz.edu.pt (A.H.S.D.); joaorua@sapo.pt (J.R.); pmonteiro@egasmoniz.edu.pt (P.M.); isantos@egasmoniz.edu.pt (I.C.S.); lproenca@egasmoniz.edu.pt (L.P.); jmendes@egasmoniz.edu.pt (J.J.M.); 3 Division of Biomaterials and Tissue Engineering, Eastman Dental Institute, University College London (UCL), London NW3 2PF, UK; 4 University Medical Center Groningen, Department of Restorative Dentistry and Biomaterials, Center for Dentistry and Oral Hygiene, University of Groningen, 9713 AV Groningen, The Netherlands; 5 Department of Special Dental Care, Martini Hospital, 9728 NT Groningen, The Netherlands; 6 Faculty of Health Sciences, University Arthuro Prat, Iquique 2120, Chile

**Keywords:** bond strength, CAD/CAM, hydrofluoric acid etching, lithium disilicate, silane treatment, surface treatment

## Abstract

There is still a lack of consensus concerning the recommended etching concentration, application time and type of silane when bonding lithium disilicate-reinforced glass ceramics manufactured by CAD/CAM. The purpose of this study was thus to conduct an in vitro study which investigates the influence of hydrofluoric acid (HF) concentration, etching time and silane type on the microtensile bond strength (μTBS) of lithium disilicate to resin composites. Thirty-nine IPS e.max CAD blocks were randomly divided between thirteen groups (*n* = 3). The variables were HF concentration (9.5 or 4.9%), etching time (20 or 60 s) and silane type (Bis-Silane, Monobond Plus and ESPE Sil Silane). The blocks were cut into beams, aged for 10,000 cycles in a thermocycler and submitted to tensile stress to determine μTBS. A control group featuring the Monobond Etch & Prime (MEP) agent that combines etching/silanisation into a simultaneous process was also added. This group was discarded from the analysis due to only having pre-test failures. The data were analysed using a three-way ANOVA (*α* = 0.05). The HF concentration, etching time and silane type significantly influenced μTBS (*p* < 0.001). Significant interactions between time and silane type (*p* = 0.004), HF concentration and silane type (*p* < 0.001) and among the three factors (*p* < 0.001) were noted. Etching lithium disilicate with 9.5% HF (60 s), followed by the application of Bis-Silane, resulted in the highest μTBS (16.6 ± 9.0 MPa). The highest concentration and etching time under study, combined with a two-part silane, resulted in the highest bond strength, while the application of MEP showed a complete pre-test failure.

## 1. Introduction

Lithium disilicate-reinforced glass ceramics are considered a strong and durable, yet esthetically minimally invasive, restorative option, since their glass matrix (in high vol%) allows them to be chemically treated and bonded to tooth substrates [1,2]. Their desirable biomechanical properties, such as high flexural strength and good fracture resistance, make them a popular choice in modern restorative dentistry [3]. This type of ceramic exhibits excellent biocompatibility and optical properties which are competitive with other ceramics, showing a 30% higher translucency than that of conventional zirconia [4,5]. Lithium disilicate ceramics can be made either by heat-pressing or in CAD/CAM milling devices and programs [6]. Lithium disilicate is considered one of the most versatile ceramic materials due to its high esthetics and good mechanical properties [7]. As such, it is widely-used in partial restorations, such as anterior veneers, posterior inlays, onlays and overlays, or in full coverage crowns and other complex fixed prostheses [4]. As such, with the aim of improving patient satisfaction by reducing the number of steps required to fabricate indirect restorations, thus saving chair time, these CAD/CAM systems are being used more often. This marks the adoption of a digital workflow philosophy which matches the technological advancements of the contemporary era [8,9].

It is essential for both the tooth and the ceramic to be surface-conditioned so that a satisfactory bond strength can be achieved, thereby increasing success rates. To do so, when lithium disilicate is considered, hydrofluoric acid etching and silane treatment are indispensable to achieve a micro-retentive surface, with high surface energy and stable chemical bonds. Such surface treatments have been established in the literature as mandatory to prepare ceramics with a high vol% of glass content [10,11,12]. However, a consensus on these protocols has not yet been reached, since etching times, concentrations and application time, or even type of silane, are highly variable among existing studies [13,14]. Clarification of these questions is therefore required.

When hydrofluoric acid (HF) is considered, it is important to understand that it is used to etch ceramics that have silica in their composition (i.e., a glass matrix), making HF useless for polycrystalline solids, such as zirconia. By creating micro-porosities that will serve as micro-retention sites, and also by increasing the energy surface tension, enhancing “wettability”, it substantially improves bond strength in a concentration and time-dependent manner [15,16]. Alternatively, silanes are subsequently used as coupling agents to achieve chemical bonds. These are bifunctional molecules containing a silicon atom (Si) bonded to three of the molecules’ alkoxy groups on one end (-Si-O-CH_3_), forming a hydrolysable ester group [17]. They contain an organofunctional methacrylate group able to react and co-polymerise with other methacrylate monomers, such as the ones present in the agent used for luting/bonding (adhesive, resin composite or luting cement). The alkoxy groups of the molecule rely on activation through hydrolysis. Therefore, they can condense, forming bonds with the silica-containing ceramic [17,18].

Complications arise when dealing with clinical choices, such as choosing the correct concentration of HF to use, which can range from 4.5 to 10% [19], or choosing the type of silane, which can come in one-part or a single bottle (pre-hydrolysed), or a two-part, non-hydrolysed system (containing a separate acid solution). Some authors found that simplified strategies resulted in poor adhesion, while others seem to favour their use [20,21]. Furthermore, deciding on the correct application time for etching or silanisation is also an extremely variable factor [13]. In fact, França et al. (2020) pointed out that there are substantial differences in chemical composition and microstructure across different lithium disilicate brands, which highlights the different etching times’ protocols [22]. Moreover, recently, some silane formulations and universal adhesive strategies also feature the functional monomer 10-methacryloyloxydecyl dihydrogen phosphate (10-MDP). As argued by some authors, 10-MDP could be favourable for increasing bond strengths in ceramic preparation protocols and could even be used as a silane hydrolyser component, due to its acidic nature, but a clear role is yet to be proven [23,24].

Thus, to elucidate on the best possible choice when surface treatments are required for bonding lithium disilicate, this study aims to evaluate the influence of HF concentration, surface etching time and type of silane used on the microtensile bond strength (μTBS) of a CAD/CAM lithium disilicate ceramic to resin composite. The null hypotheses were that (1) increasing the HF concentration (4.9 to 9.5%), (2) increasing the etching time or (3) varying the silane type do not influence the μTBS of CAD/CAM lithium disilicate-composites.

## 2. Materials and Methods

### 2.1. Materials

The materials used in this study, and their batch numbers and chemical compositions, are shown in Table 1. The samples under study were blocks of lithium disilicate-reinforced glass ceramics manufactured by CAD/CAM blocks (IPS e.max CAD, Ivoclar-Vivadent, Schaan, Liechtenstein). Thirty-nine IPS e.max blocks CAD A3-HT/C14 (12 mm × 14 mm × 7 mm) received a sintering heat treatment according to the instructions from the manufacturer (840–850 °C, Programat CS2, Ivoclar-Vivadent).

### 2.2. Sample Allocation and Preparation

**Figure 1:**
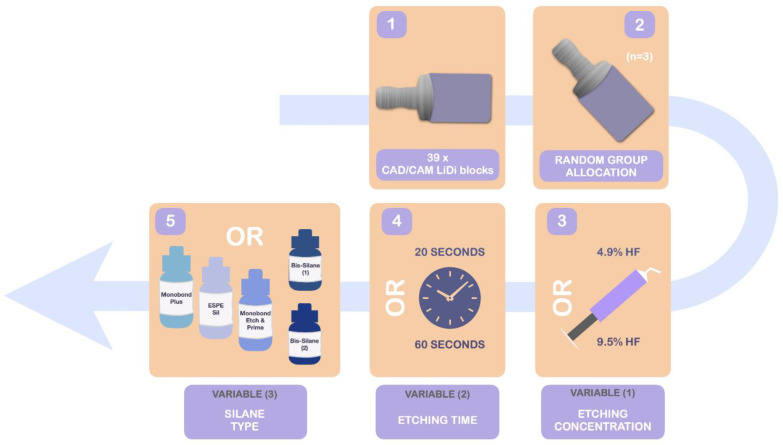
Experimental design featuring block preparation, random allocation and division according to the three variables under study.

The blocks were randomly distributed between thirteen groups (three blocks per group), according to a random number generation function (MS Excel, Microsoft, Redmond, WA, USA). Twelve groups were formed according to the experimental conditions under study: HF concentration (4.9 or 9.5%), etching time with HF (20 s or 60 s) and the type of silane coupling agent applied on the ceramic surface (Bis-Silane, Monobond Plus and ESPE Sil Silane Coupling Agent). The experimental design is shown in Figure 1.

Initially, the thirteenth group was tested using another commercial silane coupling agent—Monobond Etch & Prime (Table 1). This group was not etched with HF, nor was heat treatment of the silanised surface carried out (according to the instructions supplied by the manufacturer), since it combines both etching and silanisation into one process. This was followed by a thin adhesive layer of Optibond FL (Kerr, CA, USA), applied with a microbrush, gently air-dried for 3 s and not polymerised, in a manner equal to the other experimental groups. It was intended for comparison as a control group.

In all twelve groups, after HF etching, the samples were rinsed extensively for 20 s, and gently air-dried with an air-water syringe for 5 s. Post-etching cleaning was carried out using 35% orthophosphoric acid by actively rubbing the surface with a microbrush for 1 min (to clean the samples of extensive glass-matrix debris), then copiously rinsed with water for 20 s, gently air-dried with the air-water syringe for 5 s and finally placed in an ultrasonic bath for 5 min using distilled water. Silanisation was carried out according to the manufacturer′s instructions. For Bis-Silane, a single drop from each bottle was mixed at a 1:1 ratio, then one thin coat was applied and left to act for an additional 30 s. In the Monobond Plus group, a thin coat of silane was actively applied and allowed to act for 60 s. For the last silane system, ESPE Sil Silane Coupling Agent, a layer of silane was applied and subsequently left to dry for 5 min. After this, all of the samples (except for Monobond Etch & Prime) were heat-treated in a pre-heated oven at 100 °C for 1 min. Even though this is not specifically featured in the manufacturer instructions, the heating step was used in different studies and could enhance bond strengths [13,18]. For the consistency and reproducibility of the heat drying, an oven was used. A thin layer of the Optibond FL (Kerr, CA, USA) adhesive was applied with a microbrush for 15 s, gently air-dried for 3 s and was not polymerised.

To simulate a heated resin composite acting as a luting agent, an Enamel Plus HRi UD (Micerium S.p.A., Avegno, Ge, Italy) composite was pre-heated in an oven at 55 °C (ENA Heat Micerium S.p.A., Avegno, Ge, Italy) and packed on all specimens in increments of less than 2 mm of height, in a silicone mold. Each layer of resin was polymerised for 40 s with a high-intensity (1500 mW/cm^2^) LED unit (Elipar DeepCure-S, 3M ESPE AG, Seefeld, Germany) at a minimal distance. A pre-heated composite was chosen to bond samples as it is a restorative option used in modern adhesive dentistry, due to it having several advantages. These include vast shade choice, reduced solubility and shrinkage compared to conventional luting agents, good mechanical properties and a high degree of conversion [25,26]. The mean output intensity (>800 cm^2^) was verified regularly after every 3 exposures, using a radiometer—a Model 100 Curing Radiometer (Demetron Research Corporation, Dunbury, CT, USA).

### 2.3. Microtensile Bond Strength (μTBS) Test

The μTBS test was conducted in strict accordance with the guidelines published by the Academy of Dental Materials (2017) [27]. The samples were sectioned in a hard tissue microtome Accutom-50 (Struers A/S, Ballerup, Denmark) at low speed and under constant water cooling, to obtain beams with a cross-section of about 1 ± 0.2 mm^2^, which were then immersed in distilled water at 37 °C for 24 h before testing. A range of 50–80 beams per block were obtained. After water storage, the beams were subjected to 10,000 cycles between two water baths of 5 and 55 °C with a dwell time of 30 s in a THE-1100 thermocycler (SD Mechatronik GMBH, Feldkirchen-Westerham, Germany). The group featuring Monobond Etch & Prime had complete pre-test failures in all blocks, and as a result, was discarded before testing.

The beams were fixed to *Geraldeli*-type jigs with cyanoacrylate glue (Zapit, Dental Ventures of America, Corona, CA, USA). Each beam was tested at 0.5 mm/min until a failure in tension, in a universal testing machine using a 5 kN-load cell (Shimadzu Autograph AG-IS, Tokyo, Japan). For data analysis, all cohesive failures were discarded as they do not reflect the true value of bond strength at the interface, while pre-test failures (PTF) were included, considered as those with a microtensile bond strength value of 0 MPa [27].

### 2.4. Statistical Analysis

Data analysis was performed using IBM SPSS Statistics version 24.0 for Windows (IBM Corporation, Armonk, NY, USA), using descriptive and inferential statistics methods. Inferential analysis was carried out by using a factorial, three-way ANOVA model, considering the following fixed factors: HF concentration, etching time and silane type. Post-hoc multiple comparison analysis was performed using Tukey’s HSD test. Before the comparative analysis, the specimens’ microtensile bond strength values that were classified as outliers were removed and the factorial ANOVA model assumptions were validated. Estimated effect sizes, within the factorial model, were achieved by calculating the partial eta-squared coefficient (ƞ^2^*_p_*). The level of statistical significance was set at 5% for all inferential analyses. 

## 3. Results

The means and standard errors of the microtensile bond strength values for each of the twelve experimental groups are presented in Table 2. The Monobond Etch & Prime group had complete pre-test failures in all blocks in the aging stage, and as a result, was discarded for posterior statistical analysis and comparison.

Inferential statistics were carried out by employing a three-way ANOVA model (Table 3), which confirmed that the microtensile bond strength was significantly influenced by the HF acid concentration (*p* < 0.001), surface acid etching time (*p* < 0.001) and type of silane applied (*p* < 0.001), with estimated effect sizes (ƞ^2^*_p_*) of 0.204, 0.064 and 0.031, respectively. Additionally, significant interactions were identified among the three factors (*p* < 0.001, ƞ^2^*_p_* = 0.058), between silane type and etching time (*p* = 0.004, ƞ^2^*_p_* = 0.013) and between HF acid concentration and silane type (*p* < 0.001, ƞ^2^*_p_* = 0.045). The interaction between HF acid concentration and etching time was not found to be significant (*p* = 0.074). In order to evaluate the differences in the average microtensile bond strengths among the groups, a multiple comparison analysis was conducted. The corresponding results are expressed in Table 2, along with the descriptive statistics results for the experimental groups.

The highest average μTBS values were obtained for the group where the surface of the lithium disilicate ceramic was etched with 9.5% HF acid for 60 s, followed by application of Bis-Silane (16.6 ± 9.0 MPa). The lowest average μTBS values were obtained for the system using the ESPE Sil Silane Coupling Agent, with a 4.9% HF acid concentration and a 20 s etching time (0.6 ± 1.0 MPa).

The results of failure analysis are presented in Table 4. All specimens obtained for the Monobond Etch & Prime group had pre-test failure. For the experimental groups, the predominant fracture mode was mixed for the groups in which the ceramic surface was etched with 9.5% HF acid, ranging from 51.9 to 82.1% of total fractures. In groups where the ceramic surface was etched with 4.9% HF acid, the failure mode distribution varied strongly, depending on the silane system that was applied.

## 4. Discussion

The study confirmed the significant impact of choosing the correct concentration, etching time and type of silane, as the three variables in this study had a significant influence on the μTBS values of the ceramic–resin interface. Ageing the specimens artificially is particularly important in this study design as it is possible to mimic in laboratory settings, with the thermally-induced mechanical fatigue phenomena (due to thermal expansion) and the hydrolytic degradation in the indirect restorative bonded interface designed to last a long time [28].

Lithium disilicate-reinforced glass ceramics, according to the instructions supplied by the manufacturer, should be etched with 4.9% HF over a period of 20 s to minimise damage to the ceramic surface. However, a better surface conditioning pattern for lithium disilicate has been previously observed, with HF etching at 9.5% for 60 s, showing that it benefits from increases in time and concentration, as also confirmed in this study [29]. A study from Soares et al. (2005) also reported that HF at 9.5% for 20 s is effective in removing the second crystalline phase and glassy matrix from lithium disilicate-reinforced glass ceramics, resulting in an appropriate bonding surface which is subsequently receptive to resin composites [30]. In the present study, the microtensile bond strength results were found to be significantly higher when the ceramic surface was etched for 60 s (at a 9.5% concentration) using Bis-Silane. These results suggest that a longer etching time leads to an increased surface roughness, facilitating micromechanical retention and, consequently, higher bond strengths with adherends, agreeing with several other studies [12,31]. This concentration may not affect the flexural strength of the network [32]. In addition, as seen from the results of this study, the concentration of the etchant was the factor which had the biggest effect size, in comparison to the etching time and type of silane, implying that a greater dissolution of the glassy matrix, owing to greater etching aggressiveness, is linked to higher bond strengths. This, of course, is linked to the appropriate silane choice. These results should only be taken into account when the composition and thickness of the ceramic under use is also considered [19,22]. Chemical surface modification occurs after HF etching, with LiSiF nano-precipitates forming on the $Li_{2}Si_{2}O_{5}$ needles, having changed from a crystal morphology. These needles can then improve bonding with the silane and provide additional nano-roughness on the free surface, with a consequent densification of the three-dimensional siloxane network [33].

The choice of silane was also crucial as it directly affected the bond strength of the resulting interface. The results obtained in this study agree with those reported by Kalavacharla et al. (2015), with the highest μTBS results obtained with Bis-Silane [12]. These results can be explained by the fact that Bis-Silane is a two-part non-hydrolysed silane solution. The activation of the silane occurs upon mixing the content of the two bottles. In contrast, with pre-hydrolysed silanes, hydrolysis has already taken place since the acidic component is mixed with the silane in the same bottle, and some pre-condensation phenomena may have already occurred when the clinician applies the system. Thus, such solutions, when they are bought and used by the clinician, may contain residual silane monomers which have reacted and associated, forming oligomers that are no longer able to establish chemical bonds [17,18]. Nevertheless, pre-hydrolysed silanes are considered stable solutions even though their shelf life is considered inferior to two-component silane systems. Turning to two-component silane systems, their hydrolysis mechanisms, upon mixing, do not reach their peak in a clinically relevant application time [34].

Regarding Monobond Plus, the 10-MDP present in its composition increases the potential for chemical interactions with different surfaces. In the present study, the results obtained from the experimental groups in which Monobond Plus was applied could suggest that the addition of 10-MDP was not significant enough to compete with other silane strategies. Non-conclusive results can also be found in the literature, since some say 10-MDP may enhance the hydrolytic stability of silanes [23]. Furthermore, the acidic media created by 10-MDP is stronger in hydrolyzing silane molecules than acetic acid, forming strong siloxane bonds (-Si-O-Si) on the surface of the silica-based ceramic [35,36]. Such interactions may also result in a limitation of the self-dehydration condensation for silane molecules, through MDP hydrolysis, increasing the number of silane-ceramic bonds [24].

The lowest microtensile bond strength result was obtained in the group where ESPE Sil was applied, with an HF application at 4.9% for 20 s. This result was possibly a consequence of the pre-hydrolysis of the silane combined with the insufficient etching of the glass-ceramic. ESPE Sil is intentionally used to function as a coupling agent to silica-coated materials. In etched lithium disilicate, fewer silica-containing surfaces could be presented, and therefore its use as a silane in such circumstances could be less effective. As stated above, one major disadvantage of this type of silane is the rapid formation of oligomers without coupling ability, thus impairing the strength of the adhesive interface. The high number of pre-test failures (52.1%) in this group and its quantification at 0 MPa also contributed to the lower values for bond strength. Low values due to insufficient surface treatments provided to comparable substrates have been reported [37]. Conversely, with other silanes, failures were well distributed among the different classifications.

Monobond Etch & Prime was introduced as a single-bottle alternative to the HF etching and silane application in separate steps. It is considered a self-etching silane. It contains tetrabutyl ammonium dihydrogen trifluoride (TADF), a methacrylate phosphate monomer and a methacrylate-functionalised silane [38]. TADF is an acid salt generally used for etching silica in the surface of glass ceramics, to obtain a rough surface for micromechanical retention. TADF is a much weaker acid compared to HF, so a weaker etching pattern is expected. In fact, previous studies have proved that the resulting roughness of lithium disilicate surfaces after use of this silane, in comparison to HF etching, is substantially lower. Additionally, the authors in the same study reported that the bond strength of Monobond Etch & Prime was even lower than the negative control group after ageing [38]. Other authors also corroborated a lower roughening and etching efficacy on the surface of lithium disilicate-reinforced glass ceramics when this silane was applied, compared to traditional HF and lower shear bond strengths [39]. These results are also similar to Lopes et al. (2018) and Swank et al. (2018), which found the simplified strategy presented the lowest bond strengths and predominantly adhesive failures [20,40]. Other studies, however, observed a good adhesive performance with this ceramic conditioning agent [41,42,43]. It should be noted that, in one of the studies, the surface of the ceramic was roughened beforehand, which may have contributed to the better bond strength results, and a macrotensile setup was used as opposed to microtensile [40]. In the present study, the group in which Monobond Etch & Prime was applied may have prematurely failed due to the lack of micromechanical retention, and thus, the resulting bond strength was not enough for the samples to withstand being sectioned into beams. Micromechanical retention and surface roughness are paramount to achieve acceptable bond strengths with CAD/CAM materials [44]. Adhesion may have also been compromised, because the protocol of this new product requires the silane to be rinsed after it is applied. Moreover, following the instructions from the manufacturer, the silane heat treatment was not performed. This treatment allows for the elimination of contaminants, such as water and alcohol, that affect adhesion [18]. The heat additionally promotes the condensation reaction between silica and silane, and promotes the formation of more resistant bonds between the ceramic surface and the silane. As mentioned, it is important to stress that the use of silane shows contradictory results in the literature.

It is important to point out that silanisation is considered to be a technique-sensitive procedure. The instructions provided by manufacturers all display highly different application times and modes. Moreover, the temperature used when carrying out heat treatment demonstrates extreme variabilities in time, range and heat mode, which can range from as low as 5 s up to 2 min, performed at room temperature or heated to 100 °C. Heat treatments can also be performed in several different ways (from using air dryers to a toaster oven). As stated by Bruzi et al. (2017), an urgent need for standardisation arises, so as to inform clinicians on what the best practical approach may be in regard to silane treatment [13]. Optimal silane application is a crucial factor in guaranteeing not only immediate bond strength results, but also their stability [28]. In addition, pre-hydrolysed solutions with acidic components, such as acetic acid or phosphate group-containing monomers, may acidify over time, contributing to the instability of the system [45,46]. All of these remarks could also potentially explain the differences between the tested silanes.

A few possible study limitations should be pointed out. This study lacked a non-thermocycled group comparison because the aim was to test aged interfaces only. This, however, would have provided additional information regarding the role of hydrolysis mechanisms in different types of silane treatments in ceramic–resin interfaces, and should be carried out in the future. Moreover, heat treatment could have been carried out in the Monobond Etch & Prime group to standardise all silane groups. It is uncertain whether this would have improved its results. Nonetheless, it was decided to follow the manufacturer instructions for all tested materials.

## 5. Conclusions

The longer etching times, performed in higher concentrations, associated with two-part silanes resulted in the best approach for aged μTBS data. Specifically, etching the surface of the lithium disilicate ceramic with 9.5% HF for 60 s, followed by the application of Bis-Silane, produced the highest μTBS. The lowest average μTBS values were obtained for the ESPE Sil Silane Coupling Agent system (4.9% HF, 20 s). According to the microtensile behaviour obtained in this laboratory study for Monobond Etch & Prime, with all specimens experiencing pre-test failure, and considering all the limitations in the study design, clinical use should be cautious until further evidence is available. This study raises additional doubts concerning simplified silanisation strategies.

## Figures and Tables

**Table 1:** Materials used in the study (information as disclosed by the manufacturers).

Material	Type	Manufacturer	Batch No.	Composition
IPS Ceramic Etching Gel	Etchant	Ivoclar-Vivadent, Schaan, Liechtenstein	T29351	Hydrofluoric acid 4.9%
Porcelain Etchant 9.5%	Etchant	Bisco Inc., Schaumburg, IL, USA	1600002039	Aqueous solution of hydrofluoric acid (9.5%), 50–70% polyacrylamidomethylpropane sulfonic acid sodium fluoride
Bis-Silane	Silane	Bisco, Schaumburg, IL, USA	1600001184 1600001185	Part A: >85% ethanol, 5–10% 3-(Trimethoxysilyl) propyl-2-methyl-2-propenoic acid Part B: 30–50% ethanol, 1–5% phosphoric acid
Monobond Plus	Silane	Ivoclar-Vivadent, Schaan, Liechtenstein	V21266	50–100% ethanol, disulfit methacrylate, ≤2.5% phosphoric acid dimethacrylate, ≤2.5% 3-trimethoxysilylpropyl methacrylate
ESPE Sil Silane Coupling Agent	Silane	3M ESPE AG, Seefeld, Germany	632307	>97% ethanol, <3% 3-trimethoxysilylpropyl methacrylate, <2% methyl ethyl ketone
Monobond Etch & Prime	Silane	Ivoclar-Vivadent, Schaan, Liechtenstein	W05619	10–25% butanol, 2.5–10% tetrabutyl ammonium dihydrogen trifluoride, methacrylated phosphoric acid ester, <2.5% bis(triethoxysilyl) ethane
Optibond FL	Bonding agent	Kerr, CA, USA	6158322	Adhesive: Bis-GMA, HEMA, GDMA, CQ, ODMAB, fillers (~48%)
Resin Composite Enamel Plus HRi	Composite	Micerium S.p.A., Avegno, Ge, Italy	2017000418 2016001162 2016008171	UDMA, Bis-GMA, Butanediol, Dimethacrylate, Glass fillers, Zirconia oxide nanoparticles
Lithium disilicate ceramic IPS e.max CAD A3-HT/C14	Ceramic	Ivoclar-Vivadent, Schaan, Liechtenstein	V49313	SiO_2_, Li_2_O, K_2_O, MgO, Al_2_O_3_, P_2_O_5_, other oxides

* Percentages of components are shown in weight%. Legend: Bis-GMA: Bisphenol-A glycidyl dimethacrylate; CQ: camphorquinone; GDMA: 1,3-glycerol dimethacrylate; ODMAB: 2-(ethylhexyl)-4-(dimethylamino)benzoate; UDMA: urethane dimethacrylate.

**Table 2:** μTBS values (MPa) after 10,000 thermocycles (artificial aging) for the tested groups, presented as mean (M) ± standard error (SE). Total number of specimens (*n*). Monobond Etch & Prime was excluded from this table since this group had a complete pre-test failure, and thus its bond strengths could not be measured.

		Bis-Silane		Monobond Plus		ESPE Sil Silane CA	
HF Ac. Conc. (%)	Etching Time (s)	*n*	M ± SE	*n*	M ± SE	*n*	M ± SE
9.5	20	66	7.0 ± 0.5 ^aA^	64	10.4 ± 0.8 ^aBC^	79	12.4 ± 0.9 ^aB^
	60	76	16.6 ± 1.0 ^bA^	69	12.3 ± 0.7 ^aB^	78	12.4 ± 0.8 ^aB^
4.9	20	74	8.5 ± 0.7 ^aA^	73	4.8 ± 0.4 ^bB^	53	0.6 ± 0.1 ^bC^
	60	72	8.9 ± 0.7 ^aA^	71	6.8 ± 0.4 ^cAB^	80	5.5 ± 0.6 ^cB^

* Different lower-case letters indicate significant differences between means in the same column, and different upper-case letters indicate significant differences between means in the same row (Tukey HSD post-hoc test, *p* < 0.05).

**Table 3:** Three-way ANOVA results, considering the factors: HF acid concentration, etching time and silane type.

Source	Type III Sum of Squares	d*f*	Mean Square	*F*	*p*
Model	13,683.276	11	1243.934	35.380	<0.001
HF acid concentration	7599.039	1	7599.039	216.129	<0.001
Etching time	2017.635	1	2017.635	57.385	<0.001
Silane type	956.592	2	478.296	13.604	<0.001
HF acid concentration × etching time	112.829	1	112.829	3.209	0.074
Etching time × silane type	388.375	2	194.198	5.523	0.004
HF acid concentration × silane type	1405.889	2	702.944	19.993	<0.001
HF acid concentration × etching time × silane type	1821.657	2	910.828	25.905	<0.001
Error	29,604.431	842	35.160		
Total	113,456.851	854			

**Table 4:** Failure distribution (% within group), as a function of HF acid concentration, etching time and silane type (A—Adhesive, M—Mixed, C—Cohesive, PTF—Pre-test failure).

		Bis-Silane				Monobond Plus				ESPE Sil Silane Coupling Agent			
HF Ac. Conc. (%)	Etching Time (s)	A	M	C	PTF	A	M	C	PTF	A	M	C	PTF
9.5	20	25.0	45.0	17.5	12.5	28.4	55.2	6.0	10.4	13.6	75.3	2.5	8.6
	60	29.9	51.9	16.9	1.3	23.0	62.2	14.9	0.0	13.1	82.1	0.0	4.8
4.9	20	51.3	29.5	1.3	17.9	22.2	54.3	0.0	23.5	46.6	1.4	0.0	52.1
	60	38.5	43.6	1.3	16.7	22.2	67.9	1.2	8.6	59.0	10.8	1.2	28.9

## Data Availability

Data may be available upon request from the author.
